# Short-term high fat diet alters genes associated with metabolic and vascular dysfunction during adolescence in rats: a pilot study

**DOI:** 10.7717/peerj.11714

**Published:** 2021-07-09

**Authors:** Alex E. Mohr, Rebecca A. Reiss, Monique Beaudet, Johnny Sena, Jay S. Naik, Benjimen R. Walker, Karen L. Sweazea

**Affiliations:** 1 College of Health Solutions, Arizona State University, Phoenix, AZ, United States; 2 Biology Department, New Mexico Institute of Mining and Technology, Socorro, NM, United States; 3 National Center for Genome Resources, Santa Fe, NM, USA; 4 The Department of Cell Biology and Physiology, University of New Mexico, Albuquerque, NM, United States; 5 College of Health Solutions & School of Life Sciences, Arizona State University, Tempe, AZ, USA

**Keywords:** High fat diet, Aorta, Cardiovascular, Gene, RNA, Metabolic syndrome

## Abstract

**Background:**

Diet-induced metabolic dysfunction precedes multiple disease states including diabetes, heart disease, and vascular dysfunction. The critical role of the vasculature in disease progression is established, yet the details of how gene expression changes in early cardiovascular disease remain an enigma. The objective of the current pilot project was to evaluate whether a quantitative assessment of gene expression within the aorta of six-week old healthy male Sprague-Dawley rats compared to those exhibiting symptoms of metabolic dysfunction could reveal potential mediators of vascular dysfunction.

**Methods:**

RNA was extracted from the aorta of eight rats from a larger experiment; four animals fed a high-fat diet (HFD) known to induce symptoms of metabolic dysfunction (hypertension, increased adiposity, fasting hyperglycemia) and four age-matched healthy animals fed a standard chow diet (CHOW). The bioinformatic workflow included Gene Ontology (GO) biological process enrichment and network analyses.

**Results:**

The resulting network contained genes relevant to physiological processes including fat and protein metabolism, oxygen transport, hormone regulation, vascular regulation, thermoregulation, and circadian rhythm. The majority of differentially regulated genes were downregulated, including several associated with circadian clock function. In contrast, leptin and 3-hydroxy-3-methylglutaryl-CoA synthase 2 (Hmgcs2) were notably upregulated. Leptin is involved in several major energy balance signaling pathways and Hmgcs2 is a mitochondrial enzyme that catalyzes the first reaction of ketogenesis.

**Conclusion:**

Together, these data describe changes in gene expression within the aortic wall of HFD rats with early metabolic dysfunction and highlight potential pathways and signaling intermediates that may impact the development of early vascular dysfunction.

## Introduction

The prevalence of metabolic syndrome has increased among US adults by over 35% since 2012 and currently over two-thirds of Americans meet the diagnostic criteria (Moore, Chaudhary & Akinyemiju, 2017). Metabolic syndrome is a cluster of biological factors characterized by abdominal obesity, dyslipidemia, hypertension, and fasting hyperglycemia (Kassi et al., 2011). These factors promote cardiometabolic dysfunctions including hypertension, atherosclerosis, aging, heart and renal failure, as well as coronary syndrome (Cuspidi et al., 2018; Katsiki et al., 2014). Many of these dysfunctions are related to the vasculature and are critical accelerants of cardiovascular disease (CVD), the leading cause of death in the United States (Benjamin et al., 2019). The treatment of progressive vascular degeneration is largely hindered by vascular remodeling, which occurs during the progression from a healthy state to a diseased state (Petrie, Guzik & Touyz, 2018). In addition, lack of data on the impact of poor diet during adolescence on health as an adult and risk for CVD further complicates efforts to better delineate vascular disease progression and identification of prevention strategies (Dahm et al., 2016; Ning et al., 2015).

The pathology of vascular dysfunction is multifaceted and can be characterized as a pro-contractile, pro-inflammatory, and pro-coagulant environment at the level of the vascular wall. Molecular components of vascular dysfunction include an imbalance between endothelial-derived vasodilator and vasoconstrictor factors, increased expression of cellular adhesion molecules and pro-inflammatory cytokines, and increased expression of pro-coagulant factors (Gimbrone & García-Cardeña, 2016). Vascular dysfunction research has largely focused on protein-level signaling pathways and molecular processes. Associated gene expression changes provide invaluable insight into the genomic-level mechanisms that underlie CVD and facilitate early detection and the development of potential reversal methods for CVD.

When compared to microarray technology, RNA-Seq eliminates the need for *a priori* information necessary for designing primers, provides a less biased view of gene expression, and allows the whole transcriptome to be assayed (Rao et al., 2019). The development of post-sequencing bioinformatic workflows to effectively analyze and visualize the data is still in progress, including issues regarding the number of biological replicates and the statistical methods used for small sample sizes (Li et al., 2020). The expense of RNA-seq often requires compromising the number of replicates. RNA-seq is highly reproducible, so technical replicates, in which the same nucleic sample is subjected to sequencing multiple times, are unnecessary (Marioni et al., 2008). Statistical algorithms used for RNA-seq are designed to compensate for a low number of biological replicates and can detect differential gene expression even when no replicates are available (Maza, 2016). There are multiple software packages to accomplish the mapping and statistical analyses and it may be necessary to use different methods on a dataset to select the optimal workflow, which can include the comparison of multiple statistical methods on the same data (Kvam, Liu & Si, 2012). Additional downstream statistics, such as those used to create networks, provide biological significance to the data set. For example, statistical significance can be augmented with biological significance evaluation in the form of Gene Ontology (GO) terms enrichment tests (Chagoyen & Pazos, 2010). Biological Process (BP) terms are relevant to this study. However, a list of GO BP terms over-represented by the differentially regulated genes can be difficult to fully interpret. Network analysis can help detect the relationships between the biological processes and changes in gene expression relevant to the experiment. For example, using a workflow with two conditions, each with two replicates that consisted of pooled cells exposed to titanium of a specific grain size, gene expression differences revealed changes in biological processes associated with mechanotransduction (Reiss et al., 2020). Cytoscape and associated applications combine data-mining applications with network analysis that facilitate the visualization of data, resulting in an improved understanding of data (Bindea et al., 2009; Cline et al., 2007). Thus, examination of gene expression response in the vasculature during metabolic dysfunction provides a unique perspective on its pathophysiology and a means to identify potential molecular targets that are missed by conventional methods.

Current understanding of the early changes in gene expression leading to vascular dysfunction, especially during adolescence, remain limited. In addition, there is a paucity of studies that have examined the effects of short-term intake of a high fat diet (HFD) on gene expression within the aorta (Bao et al., 2016; Xi et al., 2017). RNA-Seq can provide important insights into gene expression and associated molecular mechanisms occurring in early stage vascular dysfunction. Therefore, the objective of the current pilot project was to demonstrate that a quantitative assessment of gene expression within the vascular wall (thoracic aorta) of healthy adolescent male rats compared to those exhibiting metabolic dysfunction could reveal potential pathways and signaling intermediates that contribute to vascular dysfunction. Specifically, we sought to identify genes differentially expressed between HFD and standard Chow-fed rats. When maintained on a HFD, these animals exhibit symptoms of metabolic dysfunction including hypertension, weight gain, glucose intolerance, and insulin insensitivity, which closely resembles the human condition (Hintze et al., 2018). Samples analyzed in the present study were obtained from rats that were part of a larger experiment (Sweazea, Lekic & Walker, 2010). Briefly, rats on the HFD had significantly greater total body mass, epididymal fat pad mass, waist circumference, fasting whole blood glucose, and systolic blood pressure in addition to impaired endothelium-dependent vasodilation and hyperleptinemia. The HFD rats exhibited symptoms of metabolic syndrome but were not yet diabetic or overtly obese. Therefore, we hypothesized that rats fed HFD would display altered expression of genes associated with vascular dysfunction and metabolic disease. To test this hypothesis, RNA isolated from the aorta of rats that were fed either a HFD or standard Chow diet (CHOW) for six weeks were sequenced and compared to detect changes in gene expression.

## Materials & Methods

### Animal experimental protocol

The male Sprague-Dawley rats (140–160 g, 5–6 weeks of age) used in this study were part of a larger investigation that compared symptoms of metabolic dysfunction in rats on CHOW and HFD treatments (Sweazea, Lekic & Walker, 2010). The composition of each diet has been described previously (Crawford et al., 2019). Briefly, the CHOW diet is the regular maintenance diet of the animals and contained in calories: 18.9% protein, 57.33% carbohydrates (% sucrose NA), and 5% fat (Cat. No. 2018; Harlan Teklad). Half of the animals were switched to a HFD after arrival that modeled a Western diet high in animal-based ingredients and saturated fat. The macronutrient profile of the HFD was 20% protein, 20% carbohydrates (6.8% sucrose) and 60% fat (in %kcal), primarily from lard (Cat. No. D12492; Research Diets, Inc., New Brunswick, NJ, USA) as described by Crawford et al. (2019). This HFD was intentionally selected to be greater than 50% fat to induce and study more severe metabolic syndrome (Rodríguez-Correa et al., 2020). In contrast, the fat content of the chow diet was plant-derived (60% soybean oil and meal, 40% wheat and corn). Similarly, the protein composition of the HFD included a mixture of casein, lactic, and 30 mesh whereas the chow diet was comprised of 36% wheat, 34% corn and 26% soy proteins. The carbohydrate composition of the chow diet included 54% wheat products, 40% corn products, and 6% soybean meal whereas the high fat diet included 63% corn and 37% sucrose.

Rats were maintained on the respective diets for 6 weeks and the food was replaced every 2–3 days to prevent spoiling. All animals had access to food and water *ad libitum* and were pair-housed in identical cages in the same animal facility and exposed to a 12:12 h light dark cycle. Animal care procedures were reviewed and approved by the Institutional Animal Care and Use Committee of the University of New Mexico School of Medicine (AAALAC # 000222). The thoracic aorta (not including the aortic arch) of eight animals, four each from the normal CHOW and HFD, were removed, cleared of surrounding fat and connective tissue, and the lumen was cleared of blood. The aortic tissue was placed in RNA later (Qiagen, Valencia, CA, USA) and frozen (−80 °C). Total RNA was extracted with the Qiagen RNeasy kit. RNA from two rats was pooled for each library (CHOW1, CHOW2, HFD1, HFD2), so four animals are represented for each treatment and were contained within two libraries. The high reliability of Illumina sequencing eliminates the need for technical replicates (Marioni et al., 2008) so each library was sequenced once.

### Library preparation and sequencing

Illumina cDNA libraries were constructed and sequenced at the National Center for Genome Resources (NCGR, Santa Fe, NW) using manufacturer’s standard operating protocols. One μg of total RNA for each sample was subjected to Illumina mRNA-SeqPrep Kit RS-100-0801 following manufacturer’s protocols. This involved two rounds of polyA selection using polyT beads, random fragmentation of RNA, random priming to make cDNA, ligation of bar-coded sequencing primers, and size selection. The bar-coded libraries were run in a single lane of a flow cell on an Illumina Genome Analyzer IIx, resulting in single-end 36 nucleotide (nt) read lengths. Raw data files (.fastq) and processed data files are available through the Gene Expression Omnibus (GEO), accession number GSE147335.

**Figure 1:**
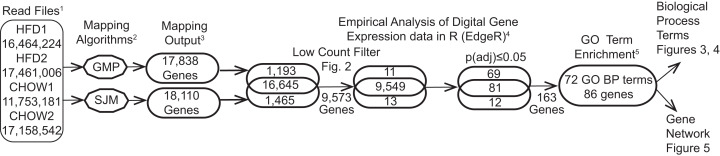
Bioinformatic workflow and results. Details of this workflow are available as supplemental file1.pdf DOI 10.6084/m9.figshare.12037272. (1) Read files are quality-control subjected files from the Illumina IIx. (2) The data was subjected to two mapping programs. (3) The results were quantitated and passed separately to edgeR. (4) The log_10_CPM data generated in edgeR was used to filter out low counts (supplemental_file_2.xlsx DOI 10.6084/m9.figshare.12037272) and select genes meeting the statistical cutoff of p(adj) ≤ 0.05 (supplemental_file_3.xlxs DOI 10.6084/m9.figshare.12037272). (5) The 163 genes meeting the statistical criteria were subjected to enrichment analysis, resulting in 86 genes that are enriched in 72 GO biological process terms.

**Figure 2:**
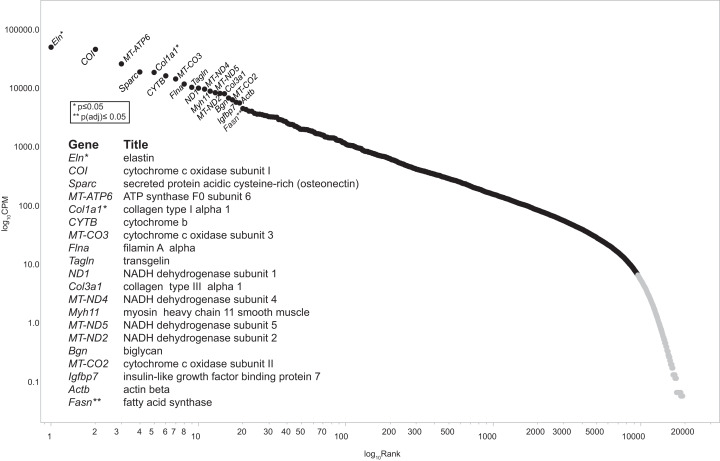
Ziph curve. By plotting the log abundance against the log rank of each gene for all samples, a line with two slopes is obtained. Genes before or near the downturn in slope (black markers) have adequate read coverage for differential expression analyses since an increase in coverage will not change the slope. This eliminates the reliance on estimates of gene length or the assumption that fold-change below an arbitrary level is insignificant. Genes with a log_10_ CPM ≥ 6.3 (about 100 reads) were selected for further analysis. This retains 9,587 genes out of a total of 19,303 (49.7%) from the consolidated dataset. An interactive version of this graph is available as Fig. S1 (DOI 10.6084/m9.figshare.12037272) Note: the file must be downloaded first and will open in browser.

The post-sequencing bioinformatic workflow is diagramed in Fig. 1. Four raw data files, two each for the CHOW and HFD condition were uploaded into Lumenogix. Data quality parameters included FASTQC (Andrews, 2010, http://www.bioinformatics.babraham.ac.uk/projects/fastqc/) and BLAST (Altschul et al., 1990) analysis of randomly selected reads. Reads were mapped to ENSEMBL release 79 of the rat genome (Rnor_5.0; GCA_000001895.3) using two methods, the Genomic Mapping and Alignment Program (GMAP) (Wu & Watanabe, 2005) and Tophat, which takes into account potential RNA splice sites, known as splice-site junction mapping (SJM) (Trapnell, Pachter & Salzberg, 2009). The results for each mapping program was quantitated per gene using HTSeq count (Anders, Pyl & Huber, 2015). The resulting files were archived in GEO (accession number GSE147335). Empirical Analysis of Digital Gene Expression data in R (EdgeR) (Robinson, McCarthy & Smyth, 2010) was used to evaluate statistical significance for the GMAP and SJM mapping methods, these results are also archived under GEO GSE147335. The resulting data files were downloaded and opened with JMP version 14 (SAS Institute, Cary, NC, USA) for data filtering and further analyses. Zipf’s power law is a graphic method employed to model complex phenomena (Adamic, 2011) that has been used to identify low count theshold in environmental metagenomic shotgun sequence data (Reiss, Guerra & Makhnin, 2016) and in a study of cell response to nanostructured titanium (Reiss et al., 2020). Transcription profiles of tissue qualifies as a complex system and a low count cutoff was determined by a Ziph graph, in which the log abundance per gene, as measured counts per million (CPM) for all samples, is graphed against the log rank of each gene (Fig. 2). The use of Ziph’s law to filter out low expressed genes replaces the application of an arbitrary threshold for gene expression. The requirement that genes be significant at *p*(adj) ≤ 0.05 in at least one mapping algorithm ensures that only those genes that are highly significant are passed onto the enrichment step.

### Data-mining and visualization with cytoscape

Genes with *p*(adj) ≤ 0.05 in either GMAP or Tophat (SJM) results were passed to Cytoscape (v3.7.1) App ClueGO/CluePedia (v2.5.5) for enrichment and network analyses. Each gene is assigned GO biological process terms (if available) and this information is used to find terms that are enriched in the data. ClueGo parameters used included a two sided-hypergeometric test with an initial cut-off of *p*(adj) 0.025, corrected with the Bonferroni step down method. Terms were limited to GO levels between 5 and 9 and the kappa score threshold was 0.4. At least three genes per term were required. The GO term fusion GO groups options were selected; these account for the redundancy of GO terms. ClueGO selects GO groups overview terms with the highest significance level (lowest *p*(adj) value). The log_2_ fold-change values were transferred into Cytoscape and displayed as gene node color. Adobe Illustrator (v24.0.1) was used for final mark-up of the networks.

## Results & Discussion

**Figure 3:**
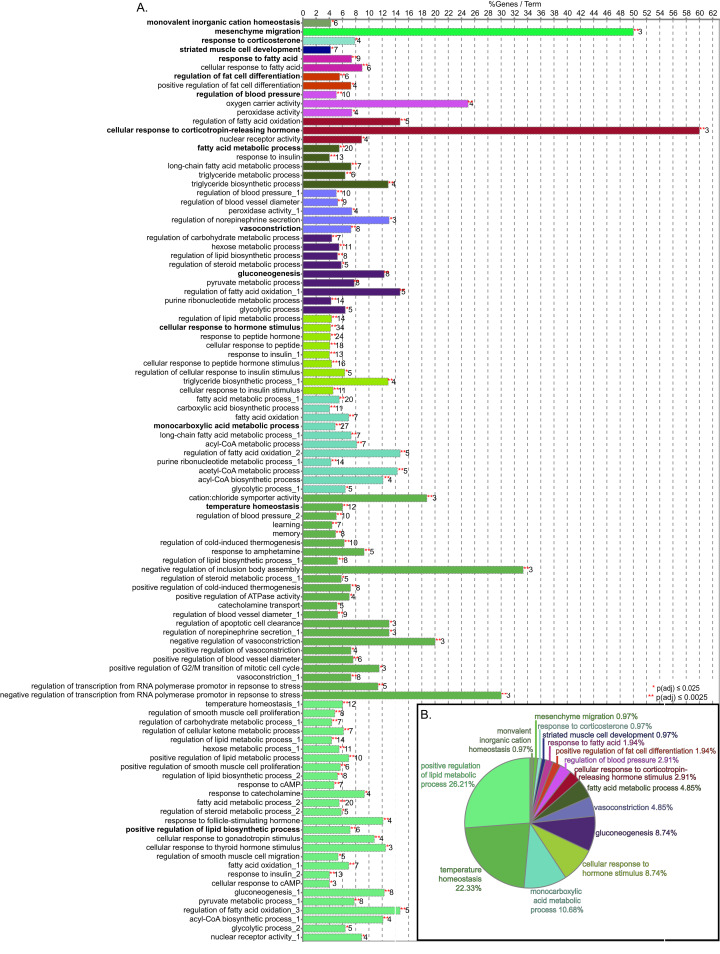
GO biological process term enrichment results. (A) Bar chart indicates the percent of genes represented in the dataset when compared to the total number of genes linked to GO term. The number of genes in this data that share the GO term shown at the end of the bar. The terms are clustered by related functions and are indicated by color, the overview term for each cluster is bolded. There are 72 terms, but some terms are in more than one cluster, these are indicated by numbers after the title. (B) Pie chart indicated the proportion of genes associated with overview terms.

The outcome of the bioinformatic workflow is shown in Fig. 1. The process started with over 62.8 million reads and ended with 86 genes with *p*(adj) ≤ 0.05 that map to 72 GO Biological process terms after enrichment analysis (Fig. 3). Of the 163 genes (Table S1, DOI 10.6084/m9.figshare.12037272) subjected to GO term enrichment, 92 (~58%) are downregulated. Of the 86 retained in the enrichment analysis, 64 (~74%) were downregulated and 22 (~26%) were upregulated in HFD compared to the CHOW condition. The level of nitric oxide synthase (NOS) showed no significant change but Leptin (Lep) was higher in the HFD rats in the physiological data reported for these animals (Sweazea, Lekic & Walker, 2010). This corresponds to the RNA-Seq data, in which *eNOS3* (endothelial nitric oxide synthase or nitric oxide synthase 3) gene shows no difference between the treatments but *Lep* is upregulated. GO BP terms were used for enrichment analysis to identify functions responsive to HFD compared to CHOW. The results of EdgeR analysis consisted of 163 differentially regulated genes with functional annotations that were subjected to GO BP term enrichment analysis using the Cytoscape App ClueGO/CluePedia (v 2.5.5). The resulting network consisted of 86 genes and 72 GO terms.

**Figure 4:**
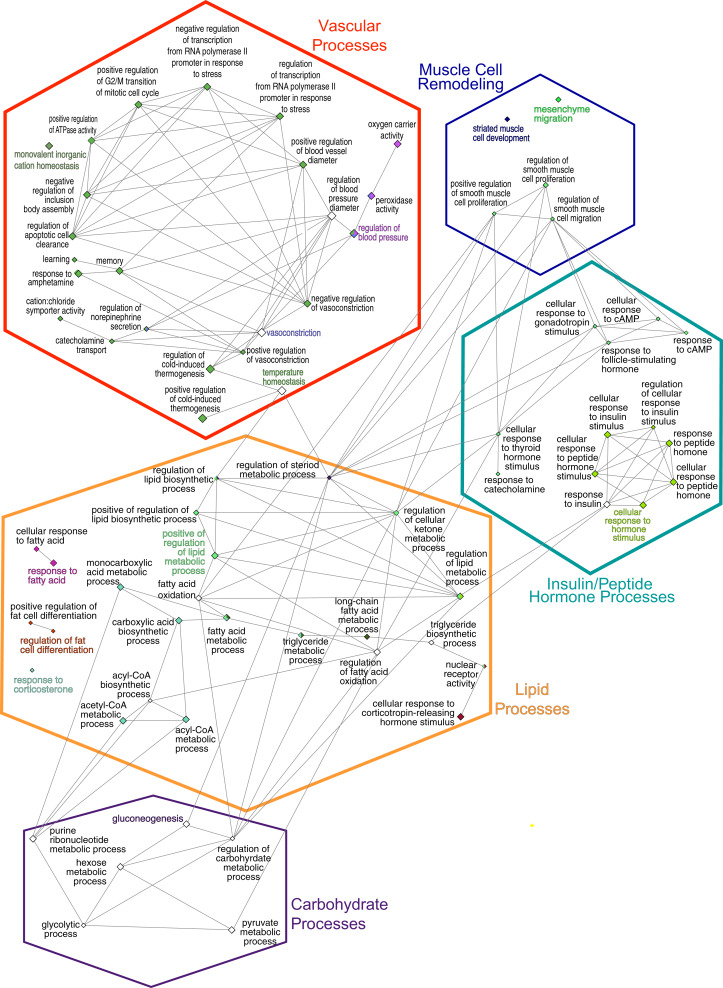
GO Biological Process network. Overview terms are based on the most significant term (Fig. 3) within the grouping and are indicated by bold color text. The edges connecting the nodes are term to term interactions. Nodes with multiple colors are assigned to multiple clusters. The size of the node indicates significance, all are *p*(adj) ≤ 0.025. Larger nodes are more significant because they are higher-level terms in the GO hierarchy, so they include more processes and hence, more genes (Fig. 3A). The hexagons are the groupings based upon the experimental protocol used in the present study and correspond to the nodes in Fig. 5.

**Figure 5:**
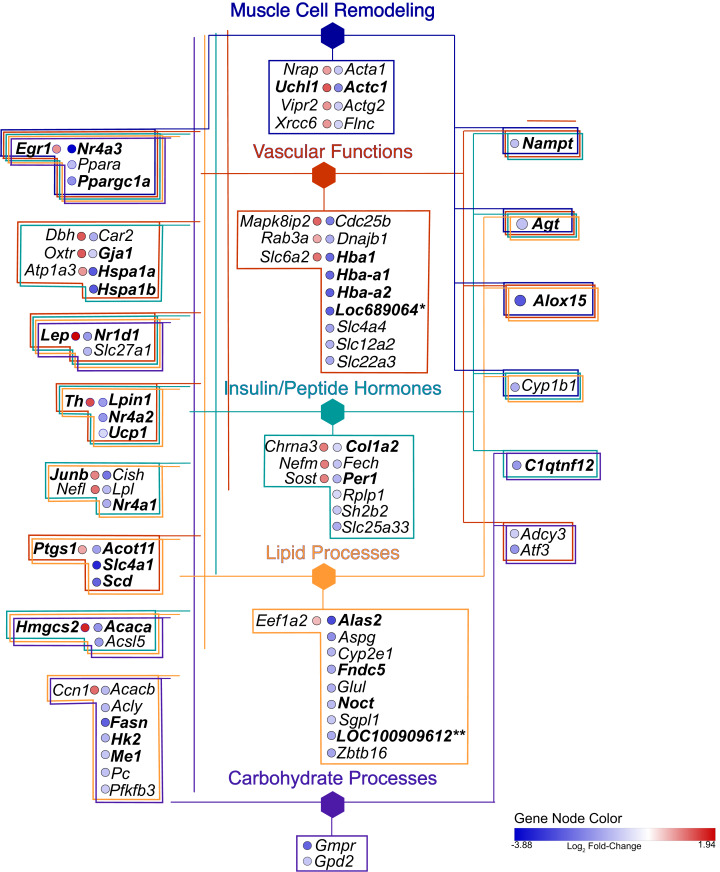
Gene network. Hexagonal nodes are the groupings shown in Fig. 4, round nodes are genes. Table 1 are the abbreviations for all genes in this network, bolded gene names are discussed in this paper. Genes in frames have are linked to the same processes. LOC287167* is an alpha-globin gene and LOC100909612** is an uncoupling-like protein.

The consolidation of GO BP terms shown in Fig. 4 is based on the experimental protocol. Five overall processes were deemed relevant to aorta, such as vascular processes and muscle remodeling. The high-fat diet treatment induced changes in multiple lipid-related processes, so these terms were consolidated. Changes in genes involved with carbohydrate processes are also correlated to the diet. Hormone signaling, especially insulin and other peptide hormones are also modulated by diet. Although there are significant GO terms that are not connected to others in the network, adding the genes to the network results in a single, interconnected network (Fig. 5). Table 1 lists all genes in the network and their fold-change in HFD aorta as compared to CHOW.

**Table 1:** Key to Figure 5 gene symbols.

Gene symbol	Title	logFC
***Acaca***	**Acetyl-CoA carboxylase 1**	**−1.6**
*Acacb*	Acetyl-CoA carboxylase beta	−1.0
*Acly*	ATP citrate lyase (Acly) transcript variant	−1.1
*Acot11*	Acyl-CoA thioesterase 11	−1.4
*Acsl5*	Long-chain-fatty-acid-CoA ligase 5	−1.5
*Acta1*	Actin alpha 1 skeletal muscle	−0.9
***Actc1***	**Actin alpha cardiac muscle 1**	**−1.8**
*Actg2*	Actin gamma 2 smooth muscle enteric	−0.8
*Adcy3*	Adenylate cyclase 3	−0.8
*Agt*	Angiotensinogen (serpin peptidase inhibitor clade A member 8)	−1.0
***Alas2***	**Aminolevulinate delta-synthase 2**	**−2.7**
***Alox15***	**Arachidonate 15-lipoxygenase**	**−2.6**
*Aspg*	Asparaginase homolog (*S. cerevisiae*)	−1.7
*Atf3*	Activating transcription factor 3	−1.6
*Atp1a3*	ATPase Na+/K+ transporting alpha 3 polypeptide	0.8
*C1qtnf12*	C1q and TNF related 12	−1.6
*Car2*	Carbonic anhydrase 2	−1.2
*Cdc25b*	Cell division cycle 25B	−2.0
*Chrna3*	Cholinergic receptor nicotinic alpha 3 (neuronal)	1.0
*Cish*	Cytokine inducible SH2-containing protein	−2.1
*Col1a2*	Collagen, type I, alpha 2	−0.6
*Cyp1b1*	Cytochrome P450 family 1 subfamily b polypeptide 1	−1.3
*Cyp2e1*	Cytochrome P450 family 2 subfamily e polypeptide 1	−1.2
*Cyr61*	Cysteine-rich angiogenic inducer 61	1.1
*Dbh*	Dopamine beta-hydroxylase (dopamine beta-monooxygenase)	1.3
*Dnajb1*	DnaJ (Hsp40) homolog subfamily B member 1	−1.2
*Eef1a2*	Eukaryotic translation elongation factor 1 alpha 2	0.5
***Egr1***	**Early growth response 1**	**0.8**
***Fasn***	**Fatty acid synthase**	**−2.4**
*Fech*	Ferrochelatase	−0.9
*Flnc*	Filamin C gamma	−0.7
***Fndc5***	**Fibronectin type III domain containing 5**	**−1.3**
***Gja1***	**Gap junction protein alpha 1**	**−0.7**
*Glul*	Glutamate-ammonia ligase	−1.2
*Gmpr*	Guanosine monophosphate reductase	−2.4
*GPD2*	Glycerol-3-phosphate dehydrogenase mitochondrial	−0.9
***Hba1***	**Hemoglobin alpha 1**	**−2.3**
***Hba2***	**Hemoglobin alpha 2**	**−2.5**
***Hbb-b1***	**Hemoglobin beta adult major chain**	**−2.5**
***Hk2***	**Hexokinase 2 (Hk2)**	**−1.2**
***Hmgcs2***	**3-hydroxy-3-methylglutaryl-CoA synthase 2 (mitochondrial)**	**1.7**
***Hspa1a***	**Heat shock 70kD protein 1A**	**−2.5**
***Hspa1b***	**Heat shock 70kD protein 1B**	**−2.5**
*Junb*	Jun B proto-oncogene	0.9
***Lep***	**Leptin**	**1.9**
***LOC100909612***	**Mitochondrial brown fat uncoupling protein 1-like**	−**1.3**
***LOC287167***	**Globin alpha **	−**2.3**
*Lpin1*	Lipin 1	−1.5
*Lpl*	Lipoprotein lipase	−1.1
*Mapk8ip2*	Mitogen-activated protein kinase 8 interacting protein 2	1.2
***Me1***	**Malic enzyme 1 NADP(+)-dependent cytosolic**	−**0.9**
***Nampt***	**Nicotinamide phosphoribosyltransferase**	−**1.0**
*Nefl*	Neurofilament light polypeptide	1.1
*Nefm*	Neurofilament medium polypeptide	1.0
***Noct***	**Nocturnin**	−**1.1**
***Nr1d1***	**Nuclear receptor subfamily 1 group D member 1**	−**1.5**
***Nr4a1***	**Nuclear receptor subfamily 4 group A member 1**	−**1.2**
***Nr4a2***	**Nuclear receptor subfamily 4 group A member 2**	−**1.6**
***Nr4a3***	**Nuclear receptor subfamily 4 group A member 3**	−**3.9**
*Nrap*	Nebulin-related anchoring protein (Nrap) transcript variant 1	0.7
*Oxtr*	Oxytocin receptor	1.3
*Pc*	Pyruvate carboxylase	−0.9
***Per1***	**Period circadian clock 1**	−**1.1**
*Pfkfb3*	6-phosphofructo-2-kinase/fructose-2 6-biphosphatase 3	−0.8
*Ppara*	Peroxisome proliferator activated receptor alpha	−1.0
***Ppargc1a***	**Peroxisome proliferator-activated receptor gamma coactivator 1 alpha**	−**1.7**
*Ptgs1*	Prostaglandin-endoperoxide synthase 1	0.7
*Rab3a*	RAB3A member RAS oncogene family	0.6
*Rplp1*	Ribosomal protein large P1	−0.6
***Scd1***	**Stearoyl-Coenzyme A desaturase 1**	−**2.4**
*Sgpl1*	Sphingosine-1-phosphate lyase 1	−0.9
*Sh2b2*	SH2B adaptor protein 2	−0.9
*Slc12a2*	Solute carrier family 12 (sodium/potassium/chloride transporter)	−1.2
*Slc22a3*	Solute carrier family 22 (organic cation transporter) member 3	−0.8
*Slc25a33*	Solute carrier family 25 (pyrimidine nucleotide carrier) member 33	−1.3
*Slc27a1*	Solute carrier family 27 (fatty acid transporter) member 1	−1.1
***Slc4a1***	**Solute carrier family 4 (anion exchanger) member 1**	−**3.3**
*Slc4a4*	Solute carrier family 4 sodium bicarbonate cotransporter member 4	−1.4
*Slc6a2*	Solute carrier family 6 (neurotransmitter transporter) member 2	1.1
*Sost*	Sclerostin	1.0
***Th***	**Tyrosine hydroxylase**	**1.4**
***Uchl1***	**Ubiquitin carboxyl-terminal esterase L1 (ubiquitin thiolesterase)**	**1.3**
***Ucp1***	**Uncoupling protein 1 (mitochondrial proton carrier)**	−**0.6**
*Vipr2*	Vasoactive intestinal peptide receptor 2	0.8
*Xrcc6*	X-ray repair complementing defective repair in Chinese hamster cells 6	0.8

**Note:**

This table lists the gene symbols and corresponding genes that were detected in the aorta samples in addition to their log fold change (logFC). Bolded entries are genes discussed in the text.

Recent advances in bioinformatics and methodology for the study of gene expression allow the identification and validation of a growing number of gene targets involved with key biological processes. As such, the goal of this pilot study was to provide a foundation from which to identify potential pathways and signaling intermediates that contribute to vascular dysfunction during early, diet-induced metabolic disease. As hypothesized, HFD-induced metabolic dysfunction altered pro-contractile and pro-inflammatory genes and biological processes associated with vascular dysfunction. The top 20 highest expressed transcripts in all conditions are consistent with that expected for aortic tissue (Fig. 2). *Elas, Col1a1*, and *Col3a1* are highly expressed and produce extracellular matrix (ECM) proteins. *Elas* and *Col1a1* are significant at *p* ≤ 0.05, while the ECM protein transcript Col1a2 is differentially regulated at *p*(adj) ≤ 0.05; all are down regulated. Principal biological processes and gene clusters that were stimulated in aortic tissue following the short-term HFD included muscle cell remodeling, vascular functions, as well as insulin/peptide hormone, lipid and carbohydrate processes. There were also several genes associated with circadian rhythm function, including *Nr1d1*, *Per1*, *Noct*, *Nampt*, *Egr1* and *Th*, that were broadly linked to five nodes informed by enrichment analysis and by the experimental protocol. These data highlight the distinct initial gene expression profiles and alteration of associated biological processes of the aortic wall during early metabolic dysfunction. Moreover, diet during early life appears to be a strong determinant of vascular dysfunction. The physiological relevance of notable genes and their linkage to the five processes shown in Figs. 4 and 5 are discussed below.

### Muscle cell remodeling

Our analysis identified 17 (~19%) genes related to muscle cell remodeling with a number of these genes associated with processes involving the regulation of smooth muscle proliferation and mesenchyme migration. While expression of the majority of these genes were consistent with the negative alterations in vascular dysfunction, these findings were notable given the brevity of the dietary intervention, age of the animals, and time-course for muscle cell remodeling in vascular diseases. Reduced expression of *Actc1* parallels findings that occur during the onset of heart disease through induction of cardiomyocyte apoptosis (Jiang et al., 2010) and increased expression of *Uchl1*, part of the ubiquitin-proteasome system associated with muscle atrophy in diabetic rats (Reddy et al., 2018). Further, *Nr4a3* was downregulated, which is a transcription factor connected to all five of the main biological functions included in our analysis. *Nr4a3* is a transcriptional target of p53, triggering apoptosis and playing a suppressive role in cancer (Fedorova et al., 2019). While the aorta was analyzed in the present research, previous studies in rats suggest the decreased expression of *Nr4a3* may be associated with reduced signaling for metabolic capacity in muscle (Oita et al., 2009; Pearen et al., 2012).

### Vascular functions

Genes associated with vascular function were abundant and included terms in the GO biological process network related to the regulation of blood pressure, vasoconstriction, temperature homeostasis, regulation of apoptotic cell clearance, and oxygen carrier activity (Figs. 3 and 4). In addition, many of the genes associated with monovalent inorganic anion homeostasis were downregulated, including *Slc4a1*. This encoded protein mediates an electroneutral chloride/bicarbonate exchange in physiological conditions and is the main membrane protein in vertebrate erythrocytes (Barneaud-Rocca, Etchebest & Guizouarn, 2013). In addition, Slc4a1 plays a role in signaling aged cells for clearance through macrophage-mediated phagocytosis (Badior & Casey, 2018). Relatedly, genes involved in oxygen carrier activity displayed reduced expression, including *Hba1*, *Hba-a2*, *Hbb-b1, and Loc689604*, a novel hemoglobin alpha gene. Recent studies in human and mice tissue have found hemoglobin alpha subunit expression in blood vessel walls, where it appears to localize at the myoendothelial junction and plays a role in regulating nitric oxide signaling between endothelium and smooth muscle (Butcher et al., 2014). Decreases in associated gene expression in the current study is suggestive of vascular dysfunction, perhaps through reduced nitric oxide signaling. This finding is corroborated with previous physiological data from these HFD animals, that exhibited impaired endothelium-dependent vasodilation (Sweazea, Lekic & Walker, 2010; Sweazea & Walker, 2011).

*Egr1* functions as a master transcriptional switch activated by ischemia to trigger expression of pivotal regulators of inflammation, coagulation, and vascular hyperpermeability (Silverman & Collins, 1999; Yan et al., 2000) and was upregulated. Although ischemic tissue damage was not directly assessed in the present study, *Erg1* activation is a notable finding considering its central role in the pathogenesis of vascular dysfunction (Yan et al., 2000). Therefore, this finding may be suggestive of early tissue damage occurring in the aortic wall of the HFD animals. Another intriguing finding was the decreased expression of *Nampt* (aka *Visfatin*). *Nampt* is the rate limiting component in the mammalian nicotinamide adenine dinucleotide (NAD) biosynthesis pathway and connects NAD-dependent sirtuin signaling, constituting a strong intrinsic defense system against various stresses (Wang & Miao, 2015). In the present study, we show a significant decrease in *Nampt* expression in aortic tissue potentially implicating this gene as nutrient-sensitive to the HFD treatment. Together the expression profile of vascular function related genes highlight potential negative alterations occurring in aortic tissue during the early stages of metabolic dysfunction, which are consistent with the impaired endothelium-mediated vasodilation observed in prior studies of ex vivo small resistance arteries from these animals (Sweazea & Walker, 2011; Sweazea & Walker, 2012).

### Insulin/peptide hormones

The adipokine encoding gene, *Lep*, displayed increased expression paralleling the elevated leptin levels in an obesogenic state (Pan, Guo & Su, 2014). In rats, leptin has been reported to stimulate the proliferation and migration of vascular smooth muscle cells in the aorta, implying leptin may play a role in the formation and development of vascular remodeling (Oda, Taniguchi & Yokoyama, 2001). Leptin appears to directly stimulate cellular hypertrophy via p38 MAP kinase in rat vascular smooth muscle cells (Shin et al., 2005). In fact, leptin receptors are present in endothelial cells providing a target for leptin action on the vascular wall (Fortuño et al., 2002).

*Hspa1a* and *Hspa1b* encode for heat shock proteins that are major orchestrators of the cellular stress response associated with several human diseases (Daugaard, Rohde & Jäättelä, 2007; McCallister et al., 2015; Richter, Haslbeck & Buchner, 2010). Both are down regulated in the HFD samples. *Hspa1a* overexpression protects many cell types against a wide variety of stresses, including hypoxia and reoxygenation injury (Hutter et al., 1994; Radford et al., 1996). Indeed, elevated Hspa1a has been reported to provide cardioprotection against ischemia-reperfusion induced myocardial infarction and apoptosis (Quindry et al., 2007). Whereas low levels of intracellular and circulating Hspa1a promote a proinflammatory state and increase the vulnerability of the arterial wall to the damaging action of vascular risk factors involved in endothelial dysfunction—the first stage in the development of the atherosclerotic plaque (Dulin, García-Barreno & Guisasola, 2012). The role of *Hspa1b* in the vasculature is less apparent and, although closely related to *Hspa1a*, it is distinct and has remained conserved in a lineage preceding placental mammals (Hess et al., 2018). Therefore, selection may be acting to preserve not only the primary function but also other less well-studied secondary functions of the molecules, like lipid binding and cell signaling (Hess et al., 2018). *Gja1* (connexin) was downregulated and similar to *Hspa1a* and *Hspa1b* is associated with both vascular functions and insulin/peptide hormones. *Gja1* encodes the protein connexin 43, a gap junction protein important in cell-to-cell communication and in vascular myoendothelial gap junctions in the heart and other tissues (Triggle et al., 2012; Xu et al., 2017). In diabetic vascular disease where blood glucose levels are both elevated and not well controlled, advanced glycation end products (AGEs) may also impact both the expression and function of connexins, and thus, contribute to the development of endothelial dysfunction and vascular disease (Wang et al., 2011).

### Lipid processes

Similar to the pattern noted with insulin/peptide hormone process, there was an expected decrease in genes encoding for enzymes involved in fatty acid biosynthesis. Of these, *Fasn* and *Acaca* encode enzymes in the fatty acid synthesis pathway and are well-known to increase with rising insulin levels associated with elevated carbohydrate intake (Saltiel & Kahn, 2001; Wong & Sul, 2010). Beyond the high level of saturated fat and low carbohydrate consumed by the HFD rats in the present study, the downregulation of these genes may also be related to increased oxidative stress, which has been noted to down regulate intermediates in the insulin signaling pathway and promote insulin resistance (Bloch-Damti & Bashan, 2005; Fridlyand & Philipson, 2006; Hurrle & Hsu, 2017). In relation, the HFD rats showed significant increases in circulating thiobarbituric acid reactive substances (TBARS) as well as vascular oxidative stress as measured by DCF fluorescence (Sweazea & Walker, 2011). In addition, they developed significantly impaired vasodilation in the mesenteric arteries that was mediated by oxidative stress (Sweazea & Walker, 2012).

Another important finding was the decreased expression of genes associated with brown adipose tissue (BAT). *Fndc5* gene was reduced in HFD rats. The encoded protein from *Fndc5* is a precursor to irisin which reportedly acts on white adipose cells to stimulate uncoupling protein expression and brown-fat-like development (Boström et al., 2012). In relation, we report decreased expression *Ucp1* and of LOC100909612, annotated as encoding a potential Upc1-Like protein. Also decreased was *Acot11*, which catalyze the hydrolysis of fatty acyl-CoA thioesters and is enriched in BAT (Cohen, 2013). *Acot11* also appears to regulate the availability of substrates for beta-oxidation and uncoupling (Han & Cohen, 2012; Okada et al., 2016; Zhang et al., 2012). While there is a reduction in BAT in humans with increased obesity and metabolic dysfunction (Franssens et al., 2017), the decreased expression of these BAT genes in the aortic wall from the present study was an unexpected finding. This may have been due to remnants of perivascular adipose tissue (PVAT), the adipose tissue surrounding blood vessels, in our tissue samples. PVAT shares several of the defining characteristics of BAT, including its cellular morphology and expression of thermogenic genes characteristic of brown adipocytes (Hildebrand, Stümer & Pfeifer, 2018). Moreover, PVAT is the source of several paracrine factors that regulate vascular tone (Chang et al., 2012; Friederich-Persson et al., 2017).

### Carbohydrate processes

There was a general decrease in genes related to the regulation of carbohydrate metabolic processes including *Hk2*, which phosphorylates glucose to produce glucose-6-phosphate and is regulated in part by insulin (Osawa et al., 1995; Okada et al., 2016). Development of diabetes is associated with a shift in cardiac glucose metabolism including significant decreases in *Hk2* content (Calmettes et al., 2015). The general downregulation in the insulin signaling pathway is notable as HFD rats display several features of metabolic dysfunction including hyperglycemia, impaired glucose tolerance, oxidative stress as well as oxidative-stress mediated vascular dysfunction (Sweazea, Lekic & Walker, 2010; Sweazea & Walker, 2011; Sweazea & Walker, 2012). Because the vasculature is located at the interface between blood and tissue, vessel walls are particularly exposed to this oxidative stress and may experience negative alterations in insulin signaling before other tissues (Wiernsperger, 2003).

### Other considerations

In the present study, the number of genes associated with circadian function is suggestive of biological crosstalk between circadian rhythm and metabolism in the vasculature. Specifically, there was a notable decrease in expression of genes related to circadian function, including *Nr1da1* (a.k.a. Rev-ERBA), *Per1*, *Noct*, and *Nampt*. In addition, the increase in *Lep* mentioned previously is intriguing given that leptin has been reported to affect circadian rhythm in mammals (Prosser & Bergeron, 2003). The relationship between the circadian clock and these genes along with their related metabolic pathways may represent a novel target for studying the pathogenesis of vascular diseases. Disruption of daily rhythms with a chronic HFD in mice has been found to be rapidly reversible in liver tissue (via PERIOD2:LUCIFERASE bioluminescence rhythm) (Branecky, Niswender & Pendergast (2015)). Though, it is unclear if this would be similar in the vasculature. In relation, time-restricted feeding in alignment with light and dark cycles has been found to partially restore day-night differences in cardiac remodeling, triglyceride synthesis, lipidome, and metabolic flexibility in mice (Mia et al., 2021). Therefore, future work should investigate HFD cessation, as well as time-restricted feeding models on circadian-related genes in the vasculature. Though this would be complex as the number of animals required, the appropriate time series of sample collection, and selection of vasculature tissue location throughout the cardiovascular system is not fully apparent. Indeed, circadian clock components display differences in expression and localization throughout the cardiovascular system, which may confer nuances of circadian clock signaling in a cell-specific manner (Anea et al., 2018).

In rodents, high fat feeding has been reported to alter circadian synchronization to light (Mendoza, Pévet & Challet, 2008), and obesity reduces circadian gene expression in concert with impaired endothelial function in the aorta (Nernpermpisooth et al., 2015). Two core clock genes, *Per1* and *Nrd1d*, were downregulated in the aorta of rats on the HFD; both are transcriptional repressors. *Per1* is reportedly decreased in vascular smooth muscle in type 2 diabetic *db/db* mice (Su et al., 2012). Rev-ERBA can reversibly bind to heme, which changes its interaction with other clock components (Yin et al., 2007). In the present study, the enzyme responsible for the rate-limiting step in Heme production, *Alas1*, was downregulated. The role of heme availability in circadian signaling and metabolic dysfunction are unresolved. NAMPT catalyzes the rate limiting step in the NAD salvage pathway and is also a clock-controlled gene (Bass & Lazar, 2016). *Nampt*-dependent oscillatory production of NAD regulates oscillation of clock target gene expression by releasing the core clock component: Clock-Arntl/Bmal1 heterodimer from NAD-dependent Sirt1-mediated suppression (Ramsey et al., 2009). *Noct* is another mitochondrial enzyme also under the control of the circadian clock, it catalyzes the dephosphorylation of NADP+ and NADPH. In contrast, *Egr1* was increased. *Egr1* appears to play a potential role in the regulation of the hepatic circadian clock in response to feeding/fasting (Tao et al., 2015). The role of *Th* expression in peripheral clocks remains to be elucidated. This provides evidence for the disruption of circadian pathways in aorta are induced by HFD, but the nature of changes remains to be determined. Furthermore, it is not clear if females would respond in a similar fashion. In fact, the response may very well be different in females as the effect of HFDs on the circadian rhythm display some notable dependence on biological sex. For instance, in female mice estradiol has been reported to regulate the daily rhythms underlying diet-induced obesity (Omotola et al., 2019). Ovarian hormones in female mice provide a protective circadian mechanism, preventing disruption of daily rhythms from high-fat feeding (Palmisano, Stafford & Pendergast, 2017). Though the age of mice is an important determinant of this response since a HFD has been noted to alter circadian genes in mammary glands of female mice during puberty (Sundaram, Johnson & Yan, 2020). These mice were the same age as the adolescent male rats used in the present study (6-weeks), therefore, future work should consider female animals in the context of puberty, as well as menopause. Such research may better delineate the putative role of estrogen as a protective agent against HFD-induced circadian disruption.

The current study has some limitations. Due to the nature of tissue extraction and the limited number of samples, it was not possible to assess time-related changes in gene expression leading to vascular dysfunction. Therefore, significant changes may have occurred at earlier or later time points. Another important aspect relates to the HFD diet used in the present rodent model. These rats were not genetically prone to develop atherosclerotic lesions, therefore the diet is likely unable to induce significant vascular disease. However, our previous work showed impaired function in these animals (Sweazea, Lekic & Walker, 2010; Sweazea & Walker, 2011). A third limitation of the present study was the small sample size in each group, although this is typical of sequencing studies of this nature as evidenced by similar sample sizes reported in other research (Bao et al., 2016; Xi et al., 2017). As a pilot study, this work is intended to inform future research efforts in this area, with larger samples. We acknowledge that the generated statistics and described effects could be within normal biological variation and need to be replicated with larger scaled research. Such research has been performed in other animal models, including a 12-month HFD study in adult pigs (Milenkovic et al., 2020), however this study and others investigating the vasculature have used microarray gene assessments (Bao et al., 2016). The technological platform employed is not trivial as microarray analysis may induce a more biased view of gene expression and does not allow the whole transcriptome to be assayed in comparison to RNA-seq (Rao et al., 2019). Importantly, the study was able to demonstrate that RNA-seq has the sensitivity to detect differences in gene expression in the given the experimental protocol. Another point to emphasize is that this data validates several findings established in the physiological literature (e.g., involvement of circadian clocks) and experimentally (e.g., the increased circulating leptin levels, hypertension and impaired vasodilation previously observed in these animals). It is important to note that gene expression data is a proxy for active protein concentrations. As such, there are four principles of data mining using GO terms that are important to understand its potentials and weaknesses. First, differential gene expression data is a direct measure of events in the nucleus. Although an mRNA template is required to produce proteins, it is subjected to post-transcriptional control and subsequent proteins require post-translational modifications, so mRNA-based assays are a proxy for fully active protein. Second, transcripts from the same gene may code for multiple proteins, some of which act in a tissue-specific manner. An example of this in Figs. 3A and 4 are the inclusion of the GO terms ‘memory’ and ‘learning;’ these terms reflect the role in the nervous system. GO term enrichment alone does not take into account the tissue and experimental protocol. Third, by their nature, gene ontologies are complex, and this is reflected by the redundancy in networks of GO terms. For example, vasoconstriction is an overview term that 4.85% of the 86 genes share, and both the negative and positive regulation of vasoconstriction are included as GO BP terms since these processes involve the same genes. The inclusion of multiple levels of GO terms (in this case 5–9) is necessary to capture an optimized subset of genes. Thus, any conclusions regarding specific gene products will need to be empirically validated.

## Conclusion

The current body of literature has placed emphasis on examining the vasculature in models of advanced metabolic dysfunction. However, by the time of diagnosis, individuals may already demonstrate a significant vascular burden and disease progression that may limit treatment options. In this pilot study we sought to identify the early effects of HFD on vascular gene expression in the aorta of male adolescent rats using an expansive RNA-Seq approach. Overall, the expression profiles of HFD compared to CHOW displayed genes and pathways that are known to be associated with metabolic derangement and vascular dysfunction. These include processes involving vascular function and muscle remodeling, which are directly relevant to aorta. In addition, the high-fat diet treatment induced changes in multiple lipid-related processes as well as genes involved with carbohydrate processes. Hormone signaling, especially insulin and other peptide hormone-related genes also appeared to be modulated by HFD. Uniquely, several genes involved in BAT development and circadian rhythm displayed altered expression, suggestive of negative alterations in these processes. In conclusion, these results contribute to our molecular understanding of the development of vascular dysfunction in the aorta as a result of a fat-rich diet.

## Additional Information and Declarations

### Data Availability

The following information was supplied regarding data availability: The raw data are available at the Gene Expression Omnibus (GEO): GSE147335.
